# Robust Extracellular pH Modulation by *Candida albicans* during Growth in Carboxylic Acids

**DOI:** 10.1128/mBio.01646-16

**Published:** 2016-11-15

**Authors:** Heather A. Danhof, Slavena Vylkova, Elisa M. Vesely, Amy E. Ford, Manuel Gonzalez-Garay, Michael C. Lorenz

**Affiliations:** a Department of Microbiology and Molecular Genetics, McGovern Medical School, University of Texas Health Science Center at Houston, Houston, Texas, USA; b The Graduate School of Biomedical Sciences, University of Texas Health Science Center at Houston, Houston, Texas, USA; c The Brown Foundation Institute for Molecular Medicine, University of Texas Health Science Center at Houston, Houston, Texas, USA

## Abstract

The opportunistic fungal pathogen *Candida albicans* thrives within diverse niches in the mammalian host. Among the adaptations that underlie this fitness is an ability to utilize a wide array of nutrients, especially sources of carbon that are disfavored by many other fungi; this contributes to its ability to survive interactions with the phagocytes that serve as key barriers against disseminated infections. We have reported that *C. albicans* generates ammonia as a byproduct of amino acid catabolism to neutralize the acidic phagolysosome and promote hyphal morphogenesis in a manner dependent on the Stp2 transcription factor. Here, we report that this species rapidly neutralizes acidic environments when utilizing carboxylic acids like pyruvate, α-ketoglutarate (αKG), or lactate as the primary carbon source. Unlike in cells growing in amino acid-rich medium, this does not result in ammonia release, does not induce hyphal differentiation, and is genetically distinct. While transcript profiling revealed significant similarities in gene expression in cells grown on either carboxylic or amino acids, genetic screens for mutants that fail to neutralize αKG medium identified a nonoverlapping set of genes, including *CWT1*, encoding a transcription factor responsive to cell wall and nitrosative stresses. Strains lacking *CWT1* exhibit retarded αKG-mediated neutralization *in vitro*, exist in a more acidic phagolysosome, and are more susceptible to macrophage killing, while double *cwt1Δ stp2Δ* mutants are more impaired than either single mutant. Together, our observations indicate that *C. albicans* has evolved multiple ways to modulate the pH of host-relevant environments to promote its fitness as a pathogen.

## IMPORTANCE

The fungal pathogen *Candida albicans* is a ubiquitous and usually benign constituent of the human microbial ecosystem. In individuals with weakened immune systems, this organism can cause potentially life-threatening infections and is one of the most common causes of hospital-acquired infections. Understanding the interactions between *C. albicans* and immune phagocytic cells, such as macrophages and neutrophils, will define the mechanisms of pathogenesis in this species. One such adaptation is an ability to make use of nonstandard nutrients that we predict are plentiful in certain niches within the host, including within these phagocytic cells. We show here that the metabolism of certain organic acids enables *C. albicans* to neutralize acidic environments, such as those within macrophages. This phenomenon is distinct in several significant ways from previous reports of similar processes, indicating that *C. albicans* has evolved multiple mechanisms to combat the harmful acidity of phagocytic cells.

## INTRODUCTION

Hematogenously disseminated candidiasis remains a critical clinical problem in hospitalized patients, causing 10 to 12% of all cases of nosocomial bloodstream infections (1–3). Risk factors include hematological malignancies, chemotherapy, and organ transplantation; even mild iatrogenic interventions, such as the use of venous catheters, can increase the incidence of candidiasis (4, 5). Dysfunctions of both the phagocytic and nonphagocytic barriers of the innate immune system predispose a patient to disseminated disease, and hence, there has been significant interest in elucidating the mechanisms by which *Candida* species interact with relevant host cell types, such as macrophages, neutrophils, endothelial cells, and epithelial cells. Understanding the molecular details of these interactions will be necessary for the development of next-generation diagnostics and therapies to improve the current mortality rate, which has remained intractable at ~40% for several decades (6, 7).

Roughly half of disseminated candidiasis cases are due to *Candida albicans* (7). This species elaborates an impressive array of virulence factors that mediate its interaction with the host, including robust hyphal growth, secreted proteases, lipases, phospholipases, and superoxide dismutases, cell surface adhesins, and proteins that bind complement regulators (8–10). Many of these factors are strongly induced following contact with host cells, particularly phagocytes. Following phagocytosis by macrophages, *C. albicans* manipulates phagosomal maturation and acidification and inhibits nitric oxide production (11–14). These virulence traits are frequently absent or more limited in closely related but less virulent *Candida* species (15). Accordingly, *C. albicans* mutant strains impaired in these host-associated traits are often less virulent, though genetic redundancy sometimes makes this hard to demonstrate conclusively.

Phagocytosis induces dramatic transcriptional changes in *C. albicans*, with the biggest component being a massive change in carbon metabolism in these cells (16, 17). We and others have shown that the catabolic pathways for a variety of nonsugar compounds are induced by phagocytosis and are required for fungal survival in macrophages and/or virulence in a mouse model of disseminated candidiasis (18–23), suggesting that certain host niches are carbon-limited environments.

Among the nonglucose nutrients potentially present in abundance in the host are fatty acids, *N*-acetylglucosamine (GlcNAc), carboxylic acids such as lactate, amino acids, peptides, and proteins; indeed, many amino acid auxotrophs of both *C. albicans* and *Candida glabrata* are fully virulent in mice, suggesting a ready supply of these compounds. We have shown that *C. albicans* can avidly use amino acids as a sole carbon source, releasing the amine groups in the form of ammonia (24). This has the effect of raising the extracellular pH, both *in vitro* and in the macrophage phagosome (14); this pH manipulation induces the hyphal morphogenesis that characterizes the *C. albicans*-macrophage interaction and is essential for fungal fitness in both tissue culture and whole-animal models (14, 25–27). Growth on GlcNAc has also been reported to result in neutralization of the medium, and mutants that cannot utilize this amino sugar are highly attenuated in mouse models, though whether this is linked to the pH phenotype has not been addressed (28). Lactate induces a number of profound changes in fungal cell wall structure, drug resistance, and immune recognition (29–31).

We report here a similar but independent phenomenon in which *C. albicans* is able to rapidly neutralize acidic environments when carboxylic acids such as α-ketoglutarate (αKG), pyruvate, or lactate are the sole carbon source. Under these conditions, cells do not excrete ammonia and do not germinate, important differences from the amino acid-driven process. Furthermore, genetic mutations that have significant phenotypes in the presence of amino acids, such as deletion of the transcription factor Stp2, do not affect growth or pH changes on carboxylic acids. Through genetic screening, we have identified several mutations that specifically block growth and/or neutralization on carboxylic acids; many of these have known phenotypes in central carbon pathways, morphogenesis, or cell wall biogenesis, one of which encodes the transcription factor Cwt1. Mutants lacking *CWT1* have a modest defect in carboxylic acid-driven alkalinization *in vitro* but occupy a more acidic phagosome and are impaired in survival following macrophage phagocytosis. We conclude that extracellular pH modulation on carboxylic acid substrates is a novel phenomenon that contributes to fungal success in host contexts.

## RESULTS

### Carboxylic acids promote rapid changes in extracellular pH.

**FIG 1:**
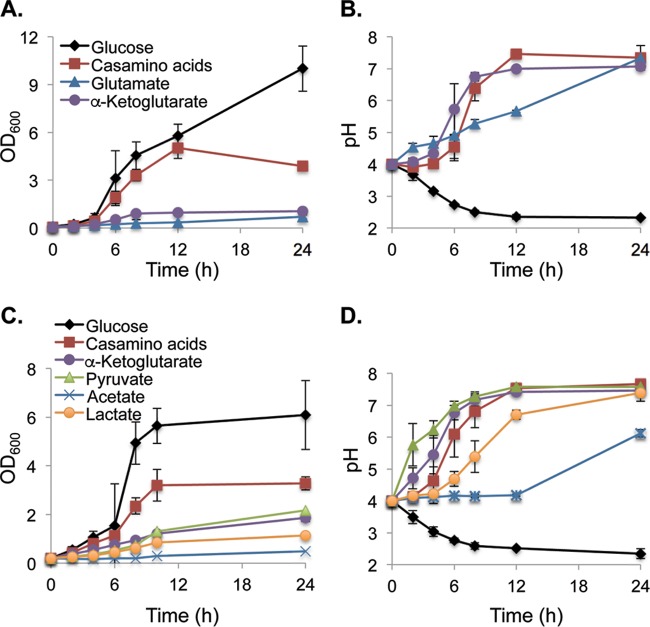
Carboxylic acids support robust extracellular alkalinization. The wild-type SC5314 strain was grown on minimal YNB medium containing 10 mM of the indicated compound as the sole carbon source at 37°C for 24 h, monitoring growth (by optical density) (A, C) and the pH of the culture medium (using a pH probe) (B, D). (A) Growth of *C. albicans* in medium with glucose, Casamino Acids, glutamate, or α-ketoglutarate. (B) pHs of the cultures whose growth is shown in panel A. (C) Other carboxylic acids also support neutralization, including pyruvate, acetate, and lactate. (D) pHs of the cultures whose growth is shown in panel C. Error bars show standard deviations.

We have proposed a model in which *C. albicans* cells growing with amino acids as the sole carbon source excrete the amine groups as ammonia, leading to an increase in extracellular pH (14, 24). This model predicts that amino acid-like compounds that lack amine groups should not produce ammonia and, presumably, would not drive changes in pH. To test our model, we grew wild-type *C. albicans* cells on minimal medium adjusted to an initial pH of 4.0 in which the sole carbon sources were glucose, Casamino Acids (CAA), the amino acid glutamate, or the deaminated form of glutamate, αKG, in which the amino nitrogen is replaced by a carbonyl group. As we reported previously, *C. albicans* cells grow rapidly in medium with either glucose or CAA (Fig. 1A); in contrast, growth was significantly slower when either glutamate or αKG was the sole carbon source. Surprisingly, all of the nonsugar compounds supported robust neutralization of the medium, including αKG (Fig. 1B). In fact, the pH rose more rapidly when αKG was the carbon source than when either CAA (slightly) or glutamate (significantly) was the carbon source. We also compared serine and pyruvate as a second pair of structurally related compounds and observed that pyruvate also promotes a more rapid rise in medium pH than does serine (data not shown).

αKG and pyruvate are C-5 and C-3, respectively, α-keto acids. We wondered whether similar carboxylic acids might also promote changes in the extracellular pH and so tested lactate (a C-3 carboxylic acid with a hydroxyl group) and acetate (a C-2 carboxylic acid). Each of these compounds could support modest growth when present as the sole carbon source under initially acidic (pH 4) conditions (Fig. 1C). As for αKG, growth on pyruvate was also associated with a very rapid rise in extracellular pH (Fig. 1D), while lactate induced a slower but still robust neutralization. Acetate is somewhat toxic at low pH, further reducing growth. Nevertheless, the pH rose by nearly two units during the course of this experiment. Thus, many carboxylic acids support growth and the modulation of the environmental pH by the fungal pathogen *C. albicans*.

### Neutralization on carboxylic acids is a physiologically and genetically distinct phenomenon.

**FIG 2:**
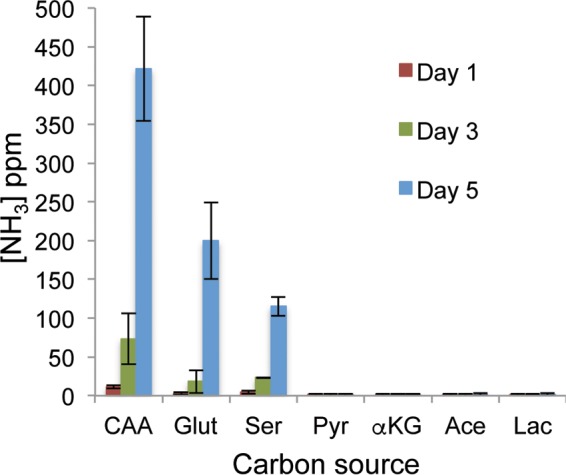
Medium neutralization induced by growth on carboxylic acids does not generate ammonia. The wild-type SC5314 strain was spotted onto solid YNB medium with the indicated compound as the sole carbon source and allowed to develop into a colony at 37°C. Directly apposed to the colony, a small reservoir was affixed to the lid of the petri dish and filled with 10% citric acid. At the indicated times, a sample of the liquid in the acid trap was removed and assayed for nitrogen content using Greiss reagent; the results are expressed as parts per million (ppm). CAA, Casamino Acids; Glut, glutamate; Ser; serine; Pyr, pyruvate; αKG, α-ketoglutarate; Ace, acetate; Lac, lactate. Error bars show standard deviations.

The data presented above might be interpreted to suggest that our model of ammonia generation through amino acid catabolism is incorrect. Alternatively, the pH changes we observe during growth on carboxylic acids may represent a phenomenon distinct from the amino acid-driven process. To address this question, we measured the amount of volatile ammonia released from cells grown on carboxylic acids. In this assay, ammonia is collected in a citric acid trap directly apposed across an air interface from a developing colony growing on solid neutralizing medium, as previously described (24). Cells growing on CAA, glutamate, or serine all excreted measurable ammonia in proportion to the speed with which they alkalinized the medium, and yet, none of the carboxylic acids lacking amine groups (pyruvate, αKG, acetate, or lactate) released any detectible ammonia (Fig. 2). This suggests that the mechanism of neutralization is distinct from that promoted by amino acids.

**FIG 3:**
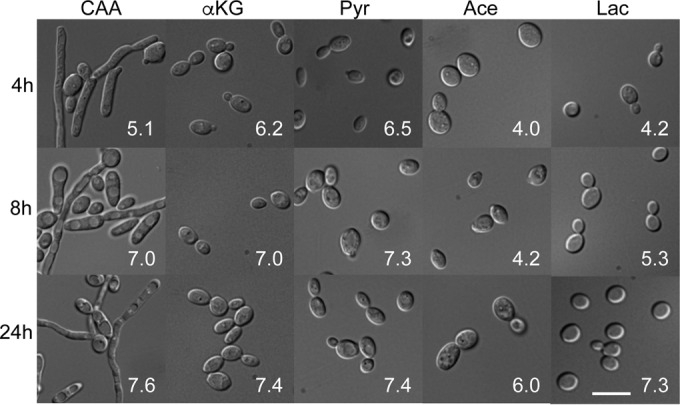
Extracellular neutralization on carboxylic acids does not induce hyphal germination. Cells of the wild type SC5314 strain were grown overnight in YPD and then washed, diluted into YNB with the indicated compound present as the sole carbon source (2% wt/vol), and grown at 37°C for the indicated times before being fixed and imaged. The number in white in each image is the pH of that culture at that time point. CAA, Casamino Acids; αKG, α-ketoglutarate; Pyr, pyruvate; Ace; acetate; Lac, lactate. The scale bar in the lowest image on the right is 10 µm.

The rise in extracellular pH during growth on amino acids induces germination (24), as neutral pH is normally a potent inducer of the switch to the hyphal form. Thus, we asked whether cells also begin to form filaments as the pH rises on carboxylic acids. To our surprise, all cells remained as yeast form throughout the experiment, despite the rapidly neutralizing pH (Fig. 3) and 37°C temperature, a combination that is usually a potent morphogenetic inducer. It is worth noting that cells grow quite slowly on these media, which might retard germ tube emergence. To address whether the carboxylic acids were inhibiting hyphal growth or amino acids were promoting it, we grew cells in medium containing CAA and αKG and observed germination similar to that with CAA alone (data not shown). To simplify this experiment, we assayed morphology in medium containing glutamate, αKG, or both. Again, αKG-grown cells remained exclusively in the yeast form, while those in medium containing glutamate began to germinate, albeit at reduced rates relative to the germination rates in medium containing CAA (data not shown). Thus, carboxylic acids do not induce germination but, also, do not inhibit hyphal formation induced by other stimuli.

**FIG 4:**
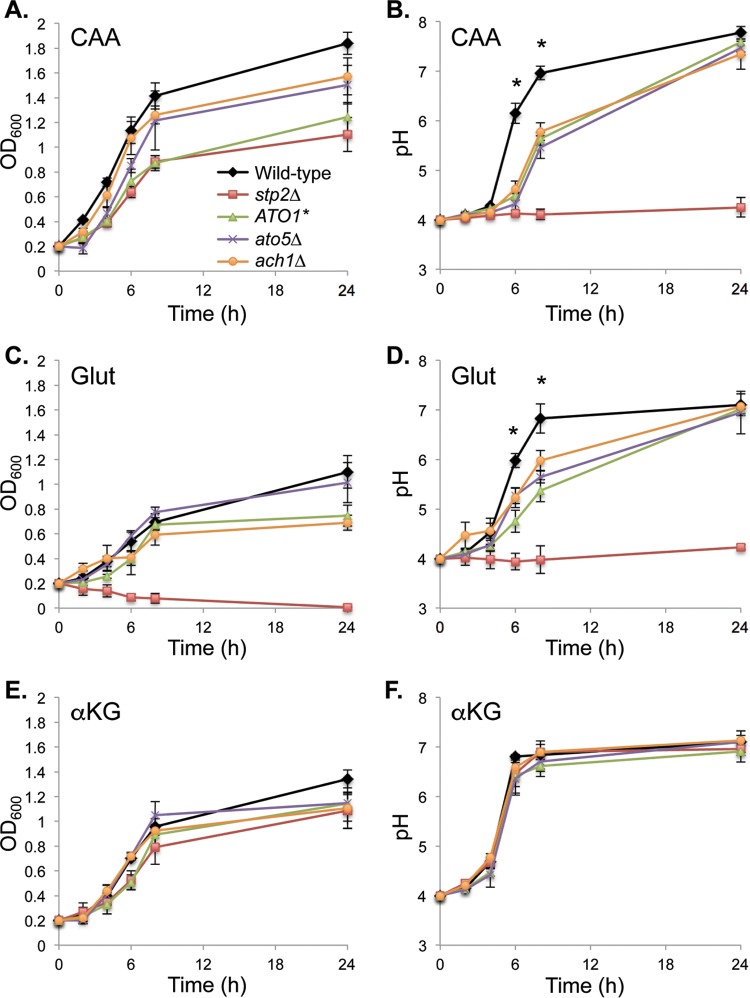
Amino acid- and carboxylic acid-driven alkalinization are genetically distinct. Strains of the indicated genotypes (Wild-type, SC5314; *stp2Δ*, SVC17; ATO1*, MLC112; *ato5*Δ, HDC31; *ach1*Δ, ACC15) were grown in minimal liquid YNB medium with the indicated carbon source: Casamino Acids (CAA) (A, B); glutamate (Glut) (C, D); or α-ketoglutarate (αKG) (E, F). Culture density (A, C, E) and pH (B, D, F) were measured at the indicated times. The culture pH for all mutants was significantly different than that of the wild-type control (*, *P* < 0.05) at *t* = 6 h and *t* = 8 h during growth in CAA and glutamate only. Error bars show standard deviations.

We and others have identified several genes required for amino acid-induced pH changes, including the genes encoding the transcription factor Stp2, a membrane sensor of amino acids (SPS, composed of Ssy1, Ptr3, and Ssy5), the acetyl-coenzyme A (CoA) hydrolase Ach1, the urea amidolyase Dur1,2, the putative acetate/ammonia transporters Ato1 and Ato5, and a putative polyamine transporter, Dur31 (14, 24, 27, 32, 33). We asked whether these mutations would similarly affect pH changes stimulated by carboxylic acids. Very similar patterns were seen during growth on both CAA and glutamate, in which the *stp2Δ* mutation completely abrogated any change in pH, while the remaining mutants we tested significantly retarded but did not eliminate neutralization (Fig. 4B and D). In contrast, none of the mutants tested affected growth or pH changes in the presence of αKG (Fig. 4E and F) or the other carboxylic acids (data not shown). Thus, the genetic control of extracellular neutralization on carboxylic acids is much different, further supporting the conclusion that these are distinct processes.

### Genomic analysis of growth on carboxylic acids.

**FIG 5:**
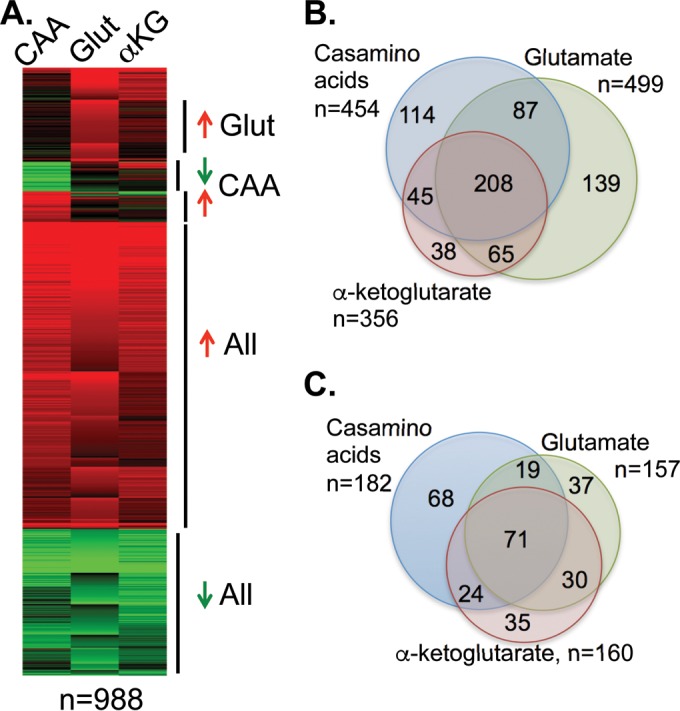
Transcriptional changes in response to amino acids or α-ketoglutarate are substantially similar. Transcriptional profiles were assessed using RNA sequencing of cells grown in minimal YNB medium containing glucose, Casamino Acids (CAA), glutamate (Glut), or α-ketoglutarate (αKG) for 5 to 7 h at 37°C. Ratios of transcript abundance based on the reads per kilobase per million (FPKM) metric under each condition relative to the expression in the glucose control were used to determine differentially regulated genes. (A) *K*-means clustering of differentially regulated genes demonstrates the similar changes under the three conditions. (B) Venn diagram of the overlap between the three conditions in genes upregulated by at least threefold. (C) Downregulated genes.

We took both genetic and genomic approaches to understanding the difference in metabolism and pH modulation during growth on amino acids versus carboxylic acids. First, we assayed transcriptional profiles using RNA deep sequencing for cells grown in minimal yeast nitrogen base (YNB) medium containing glucose, CAA, glutamate, or αKG. This approach was comprehensive, and we detected at least some level of expression for all but 32 protein-coding genes (29 nuclear and 3 mitochondrial) annotated in genome assembly 21 by the Candida Genome Database (details of the sequencing coverage and quality are in Table S1 in the supplemental material). There was a significant overlap in the sets of genes regulated by the three alternative carbon sources (relative to their expression in glucose-grown cells); for instance, 318 of the 356 genes (89%) that were induced more than threefold by growth in αKG were also induced under at least one other condition (Fig. 5A and B). A similar overlap in expression patterns was also observed in the downregulated gene sets (Fig. 5A and C).

We used *K*-means clustering to define groups of genes with similar expression patterns (Fig. 5A). As described above, the majority of these genes are coregulated under all three conditions, with smaller sets of genes differentially regulated in only one carbon source. We extracted the open reading frame (ORF) designations from these clusters and used Gene Ontology (GO) term mapping through the Candida Genome Database to identify biological processes that were overrepresented in each cluster. Table 1 lists a selected sample of these terms (there is significant overlap in GO terms, so we selected nonredundant and biologically significant terms). Enriched GO terms for coregulated genes included those for the tricarboxylic acid (TCA) cycle, fatty acid oxidation, and respiratory chain biogenesis, while terms for repressed genes included glycolysis and amino acid metabolism. While these observations were expected given previously published transcriptional profiles (16, 17), our data demonstrate a comprehensive coordination of central carbon metabolism under alternative carbon conditions, with a concerted flow of carbon toward acetyl-CoA and onward to glucose. Every step of the TCA cycle and the gluconeogenesis-specific genes is induced, while all of the glycolytic steps are repressed (see Fig. S1 in the supplemental material).

**TABLE 1:** Significantly induced GO terms (selected terms only)^a^

GO identifier	GO term	No. of genes in set of total annotated^b^	*P* value
Induced in multiple carbon sources (*n* = 352)			
6099	Tricarboxylic acid cycle	9 of 16	2.6 × 10^−5^
19395	Fatty acid oxidation	8 of 14	1.3 × 10^−4^
97031	Respiratory chain I biogenesis	7 of 13	0.0014
6083	Acetate metabolic process	7 of 13	0.0014
Repressed in multiple carbon sources (*n* = 140)			
6096	Glycolytic process	11 of 16	5.8 × 10^−13^
1901605	Amino acid metabolic process	14 of 138	6.0 × 10^−4^
9102	Biotin biosynthetic process	4 of 6	0.0013
Induced only in Casamino Acids (*n* = 38)			
1901606	Amino acid catabolic process	6 of 32	3.9 × 10^−6^
Repressed only in Casamino Acids (*n* = 46)			
6526	Arginine biosynthetic process	7 of 9	4.3 × 10^−12^
1901607	Amino acid biosynthetic process	13 of 102	2.4 × 10^−12^
97	Sulfur amino acid biosynthetic process	5 of 26	1.7 × 10^−4^
Induced only in glutamate (*n* = 96)			
42254	Ribosome biogenesis	14 of 298	0.034
3333	Amino acid transmembrane transport	5 of 40	0.092^c^

a GO terms significantly enriched in the genes identified in each category by both rank ordering and *K*-means clustering, as determined using the GO Term Finder at the Candida Genome Database. Due to extensive overlaps in GO terms, only selected terms are shown.

b Genes in the selected data set that map to that term out of the total number of genes in the genome annotated to that term.

c Though the *P* value is greater than 0.05, the false discovery rate for this term was 5.0%.

Of note, the entire fatty acid β-oxidation pathway is also induced (see Fig. S1 in the supplemental material), despite the fact that none of the media used contain any lipids. In *Saccharomyces cerevisiae*, β-oxidation is subject to regulation by Mig1, in which the absence of glucose leads to a basal derepression. However, the *C. albicans* β-oxidation genes are induced more—in some cases far more—than would be expected from glucose derepression. It is unclear why this pathway would be transcriptionally active under these conditions, though we and others have previously noted pleiotropic phenotypes of β-oxidation mutants (20, 34). This highlights a significant difference between the regulation of carbon metabolism in *C. albicans* and its regulation in other species, such as *S. cerevisiae*, as has been previously proposed (23, 35, 36).

Enriched GO terms for the gene sets induced or repressed by only a single compound are also logical given the expected changes in metabolism (Table 1). Growth in CAA, containing most of the amino acids, induces amino acid catabolism while repressing amino acid biosynthesis. Glutamate-grown cells increase their transmembrane amino acid transport capacity and, less expectedly, ribosome biogenesis. Several of the other single-condition-regulated gene sets were small and had no significant GO term enrichment.

### Genetic analysis of carboxylic acid-induced alkalinization.

Because none of our previously identified mutations conferred phenotypes for growth on carboxylic acids, we conducted a screen of two mutant libraries. The Homann library includes 166 homozygous mutations in putative transcriptional regulators (37), while the Noble library contains homozygous deletions in ~674 genes with a wide array of predicted functions (38). Both libraries contain two independently constructed mutants for most genes, increasing the robustness. To perform the screen, we transferred cells of each strain growing in rich yeast extract-peptone-dextrose (YPD) medium to 96-well plates containing YNB medium with 10 mM αKG as the carbon source, adjusted to pH 4 and containing bromocresol purple to visualize the pH. Both of these libraries are auxotrophic for arginine (*arg4*Δ), so our screening media also contained 40 µM arginine, a concentration empirically determined to be the minimal amount needed to support full growth. These were incubated at 37°C for 24 to 48 h and inspected visually for wells in which the medium remained acidic, growth was not severely impaired, and both independent mutants exhibited similar phenotypes. Secondary screens in aerated tubes validated those with significant alkalinization defects.

We identified mutations in six genes that conferred defects in medium alkalinization during growth on αKG: *ALI1, SIN3, COX4, PEP8, KIS1*, and *CPH1* (Table 2; see also Fig. S2 in the supplemental material). Mutants that entirely failed to grow were excluded from further analysis. Several of these encode proteins with functions with a clear link to carbon metabolism, such as the cytochrome *c* oxidase Cox4 and the Snf1-associated protein Kis1, while others have a less obvious connection, such as Sin3, a transcriptional repressor that promotes histone deacetylase recruitment (39), and Pep8, whose yeast homolog mediates retrograde endosome-to-Golgi vesicle transport (40). Cph1 is a well-studied transcriptional regulator of morphogenesis that is activated by the pheromone-responsive mitogen-activated protein (MAP) kinase pathway (41, 42). Cph1 was previously shown to regulate galactose utilization genes but had not otherwise been associated with metabolic functions (36). Ali1 is a recently described plasma membrane protein with roles in cell wall structure and oxidative stress responses (43).

**TABLE 2:** Mutants with pH defects in medium containing α-ketoglutarate

Gene	Growth defect		Phenotype affecting:		Function
	Glucose	αKG	Cell wall	Morphology	
*ALI1*	+	+	+	+	NADH-ubiquinone oxidoreductase
*COX4*	+	+		+	Cytochrome *c* oxidase
*CPH1*		+	+	+	Morphology transcriptional regulator
*CWT1*			+	+	Cell wall transcriptional regulator
*KIS1*		+		+	Snf1 signaling complex
*PEP8*		+		+	Retrograde vesicular transport
*SIN3*	+	+		+	Transcriptional corepressor

^a^ +, the gene, when mutated, confers either slow growth on medium containing dextrose or αKG or an aberrant phenotype related to cell wall function of morphology. Phenotypes are from this study or references 38, 43, 45, and 47.

Notably, all of these mutants confer partial growth defects on medium containing αKG (see Fig. S2 in the supplemental material). The linkage of growth and pH changes probably reflects a requirement for metabolism of these acids to effect alkalinization. Furthermore, they have all been reported to confer aberrant filamentation profiles, while several of them have altered sensitivities to agents like Calcofluor white, caspofungin, Congo red, or SDS that suggest perturbations in cell wall structure and function (38, 41–45). Several of these mutants (*ali1Δ* and *cph1Δ* mutants) are also known to be impaired in cell culture or whole-animal models of virulence (25, 43). Only *ALI1* was among the genes induced during growth on αKG relative to its expression on glucose (4.1-fold).

**FIG 6:**
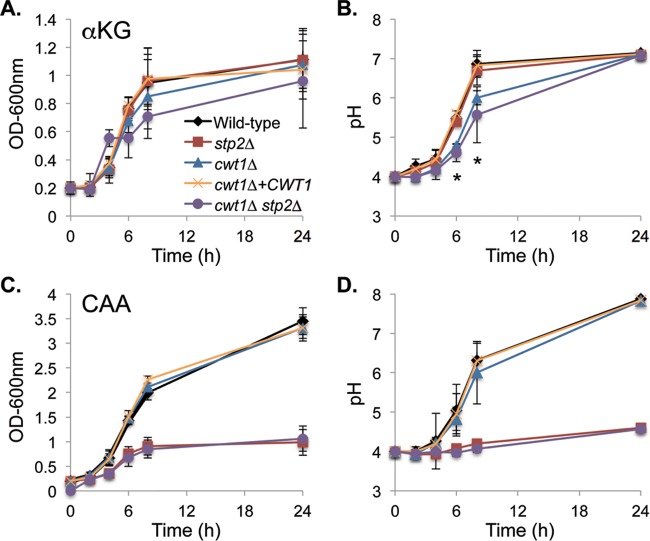
*cwt1Δ* and *stp2Δ* mutants have distinct phenotypes. Strains of the indicated genotypes (Wild-type, SC5314; *stp2*Δ, SVC17; *cwt1*Δ, SVC36; *cwt1Δ stp2Δ*, SVC39) were grown overnight in YPD and then diluted into YNB medium containing α-ketoglutarate (αKG) (A, B) or Casamino Acids (CAA) (C, D). (A, C) Growth was monitored by optical density. (B, D) pHs of the supernatants of the cultures whose growth is shown in panels A and C, respectively. (B) Significant differences (*, *P* < 0.05) were found for the *cwt1Δ* and *cwt1Δ stp2Δ* mutants relative to the WT or complemented controls at *t* = 6 h and *t* = 8 h. (C, D) The defects of the *stp2Δ* and *cwt1Δ stp2Δ* mutants in CAA medium are also significant. Error bars show standard deviations.

We reasoned that mutants with growth defects would be likely to have altered interactions with phagocytes. To separate the effects of these mutations on growth on αKG from the alkalinization phenotype, we reanalyzed our genetic data to identify mutants that might have defects at earlier time points but not at the endpoint of the assay. One such strain carried a deletion of *CWT1*, and mutants lacking this transcription factor have been reported to be sensitive to nitrosative stress and to cell wall-damaging agents and to be required for full virulence in the systemic model (46–48); in contrast, a systematic survey of transcription factor mutants reported no phenotypes for the *cwt1Δ* strain (37). To address this discrepancy, we generated an independent homozygous *cwt1Δ* mutant strain, using the SAT flipper approach (49), along with a complemented strain. As seen by the results in Fig. 6, this new mutant grows well on αKG-containing medium (Fig. 6A) and has a modest delay in neutralization (Fig. 6B). The mutation does not affect growth or pH changes when amino acids are the carbon source (Fig. 6C and D).

To test whether impairing the ability of the cell to modulate pH on amino acids and carboxylic acids is synergistic, we generated a double *cwt1Δ stp2Δ* strain by deleting *CWT1* in an existing *stp2Δ* mutant (14). This strain behaved like a *cwt1Δ* single mutant when grown on αKG (Fig. 6A and B) and like an *stp2Δ* single mutant on amino acids (Fig. 6C and D), as expected.

### Cwt1-mediated pH neutralization contributes to fungal survival in macrophages.

**FIG 7:**
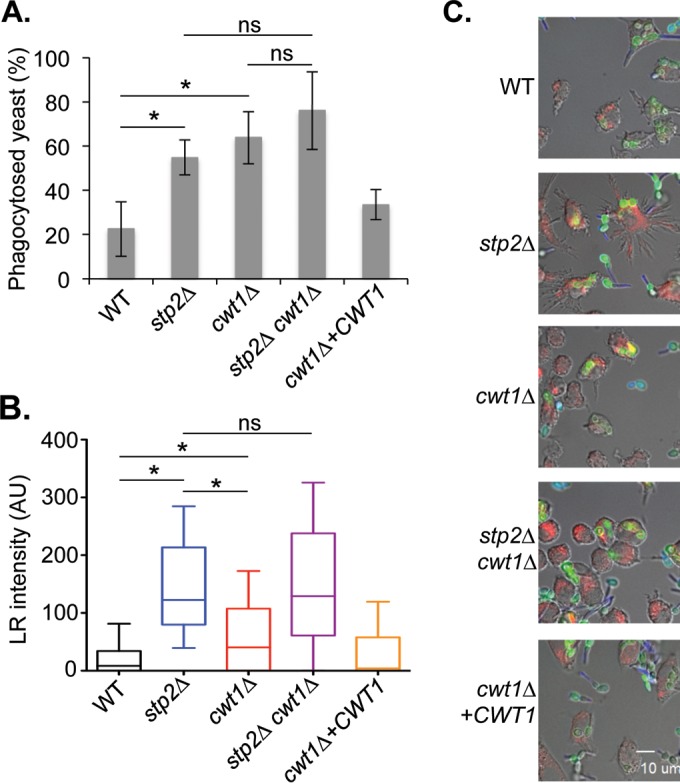
*cwt1Δ* mutants fail to germinate or fully neutralize the phagosome. (A) FITC-labeled strains of the indicated genotypes (see the legend to Fig. 6) were incubated with J774A.1 macrophages for 1 h before fixing and staining with Calcofluor white to discriminate between extracellular and intracellular *C. albicans* cells. The morphology of phagocytosed cells only was scored and plotted. Error bars show standard deviations. (B) The indicated strains were incubated with LysoTracker red (LR)-loaded J774A.1 cells for 1 h before fixation. LR intensity in the 10 pixels (1 µm) immediately adjacent to the fungal cell along a line perpendicular to the cell was averaged for at least 50 cells per strain and plotted in the box-and-whisker plot, where the whiskers show the 5th to 95th percentiles. (C) Representative images are shown in which the fungal cells are labeled with FITC (green), while the macrophages are loaded with LR (red). Calcofluor white (blue) is used to differentiate external *C. albicans* cells (which stain blue) from phagocytosed cells (which do not stain). Asterisks represent a *P* value of <0.05 for the indicated comparison.

We have reported that *stp2Δ* mutant cells, which are unable to neutralize amino acid-rich media, occupy more acidic phagosomes, germinate less readily, and are more susceptible to macrophage killing than control strains. To test whether metabolism of carboxylic acids plays a similar role, we cocultured concanavalin A-fluorescein isothiocyanate (FITC)-labeled strains with J774A.1 murine macrophages. To distinguish between phagocytosed and external *C. albicans* cells at 1 h of incubation, we stained with the membrane-impermeant dye Calcofluor white, which binds to chitin in the fungal cell wall, and then fixed the cells with paraformaldehyde. We detected no significant difference in the rates of phagocytosis of the single or double mutants relative to the rate of phagocytosis for the control (data not shown). However, it was immediately obvious that phagocytosed *cwt1Δ* or *stp2Δ* cells germinated much less readily, and this was confirmed by scoring the morphology of cells in multiple microscopic fields (Fig. 7A). The proportions of both the *cwt1Δ* and *stp2Δ* mutant cells that remained in the yeast form were similar, with a further modest increase in the double mutant. No differences in morphology were observed in nonphagocytosed cells (data not shown).

To ask whether phagosomes containing *cwt1Δ* cells differed in pH from phagosomes containing a wild-type control, we preloaded macrophages with the acidophilic dye LysoTracker red (LR) before initiating the coculture with FITC-labeled cells. After 1 h, we fixed and stained with Calcofluor and then quantitated the LR intensity surrounding the phagocytosed fungal cell (in the phagosomal lumen), as we have described previously (27). To do so, we averaged the background-subtracted LR fluorescence across the 10 pixels (1 µm) immediately outside the cell, as delineated by the FITC-labeled cell walls, along a line drawn through the short axis of the cell. This was done for at least 50 cells per strain in each of three experiments and, as seen by the results in Fig. 7B, there is a marked difference in the LR accumulations surrounding wild-type cells and *stp2Δ* mutant cells, with the mutant occupying a much more acidic compartment, as we have reported previously (14). The *cwt1*Δ strain was also found in more acidic compartments, but this effect was not as pronounced as for the *stp2Δ* mutant. The double mutant resembled the *stp2Δ* single mutant strain. Representative microscopic images are shown in Fig. 7C. Thus, Cwt1 contributes to the ability of *C. albicans* to neutralize the phagosome, but it plays a secondary role relative to Stp2.

**FIG 8:**
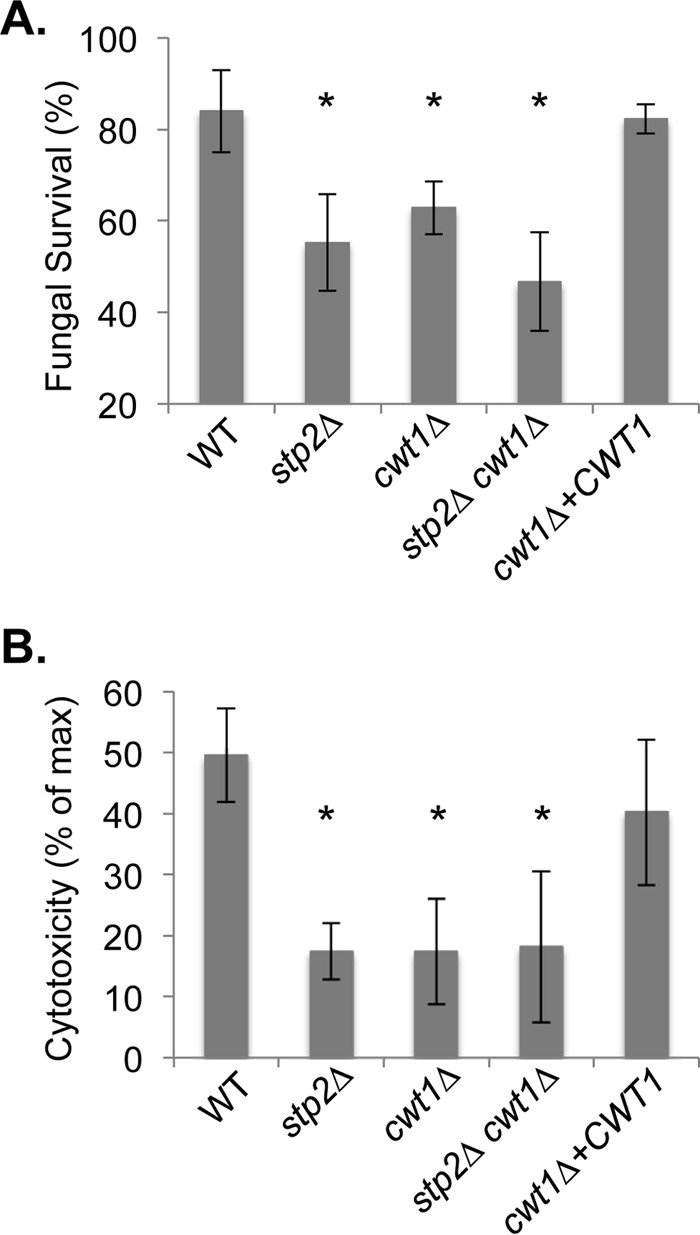
Cwt1 contributes to fungal survival in macrophages. (A) The survival of the indicated strains (see the legend to Fig. 6) was assessed using an endpoint dilution assay after coculture with J774A.1 cells for 24 h and expressed relative to the survival of cells cultured in the absence of macrophages. (B) The release of lactate dehydrogenase (LDH) from macrophages was assayed as a measure of fungus-induced membrane damage and expressed relative to the release of LDH from chemically lysed macrophages. *, *P* < 0.05 relative to the wild-type control strain. Error bars show standard deviations.

Defects in germination and modulation of phagosomal pH may compromise the ability of the cell to survive phagocytosis or to damage macrophages. To address this, we first measured the survival of fungal cells cocultured with macrophages at a very low fungal/host cell ratio using an endpoint dilution assay (14, 50). After 24 h, the difference in the number of microcolonies formed relative to the number in a macrophage-free control was used to calculate fungal survival. As seen by the results in Fig. 8A, deletion of *CWT1* compromises survival after phagocytosis to a similar extent as the *stp2Δ* mutation. A slight further reduction is observed in the double mutant, but this is not statistically significant.

Wild-type *C. albicans* cells kill macrophages both through mechanical disruption via hyphal growth and through induction of pyroptosis (51, 52). We assessed macrophage membrane damage by measuring the release of cytosolic lactate dehydrogenase into the culture medium after coincubation for 5 h, as we have described previously (14). The single *cwt1Δ* and *stp2Δ* mutants and the double mutant all significantly reduce macrophage cytotoxicity relative to the reduction by control strains, but there is no difference between the single and double mutants (Fig. 8B).

## DISCUSSION

The work presented here demonstrates that *C. albicans* avidly utilizes carboxylic acids like α-ketoglutarate, pyruvate, and lactate as the sole source of carbon and, in doing so, rapidly neutralizes the extracellular environment. Though this is superficially similar to the phenomenon we have described during catabolism of amino acids (14, 24), several lines of evidence indicate that these are unrelated activities. First, pH alkalinization on carboxylic acids does not generate ammonia, nor does it induce cells to switch to the hyphal morphology. While the transcriptional profiles of cells grown on either carbon source are quite similar, mutants that impair amino acid-driven neutralization do not inhibit the carboxylic acid phenomenon and vice versa. A genetic screen identified mutations that reduce growth and/or the ability to manipulate pH. We focused on *CWT1*, encoding a transcriptional regulator with known roles in response to cell wall damage and nitrosative stress, because it dissociated growth and pH defects. Mutants lacking *CWT1* are impaired in several aspects of the host-pathogen interaction, as are other nonalkalinizing mutants. Thus, we have identified another independent means by which *C. albicans* can manipulate the pH of the phagolysosome.

*C. albicans* uses a variety of nonglucose carbon sources far more efficiently than related nonpathogenic species, such as *S. cerevisiae*, including amino acids, fatty acids, carboxylic acids, and *N*-acetylglucosamine, all of which are present in host environments. There is accumulating evidence that cells use multiple carbon sources during growth in the host, including findings of transcriptional activation of the catabolic pathways following phagocytosis and in animals (16–18, 53–55) and attenuated virulence of mutants with mutations that disrupt gluconeogenesis, the glyoxylate cycle, or peroxisomal functions (18, 21, 34, 56, 57).

While utilization of these compounds generates energy and biomass, nonglucose carbon sources also seem to be a signal of specific host niches and result in significant changes to cellular metabolism and/or physiology. One of the most potent inducers of hyphal growth is the presence of the ubiquitous sugar *N*-acetylglucosamine (58); metabolism of this compound raises extracellular pH, but this is not required for the hyphal induction (28). Similarly, we have shown that lactate, also abundant in the host, promotes alkalinization as well. Elegant work from the Brown laboratory has demonstrated that cells grown on lactate are more resistant to stresses, including antifungal drugs, are less readily recognized by the immune system, and have altered cell walls (29–31). Our genetic analysis of growth and pH modulation on carboxylic acids reinforces the connection between alternative carbon metabolism and virulence-related processes. Each of the seven genes identified have been linked to defects in morphogenesis, and a subset of these (including *CWT1*) have aberrant cell walls that increase susceptibility to various stressors, including clinically relevant antifungal drugs (38, 41, 43–47).

While the *cwt1Δ* mutant incorporates carboxylic acids equally as well as control strains, it is retarded in neutralization of acidic media. This extends to the phagosome, where *cwt1Δ* strains occupy a more acidic compartment than wild-type strains, germinate less often, and are more readily cleared by macrophages. While our work is highly correlative, we cannot draw a definitive causal link between these phenotypes. Two possibilities exist, the first being that the inability to neutralize the phagosome is directly responsible for the impairments in macrophages and the second that the cell wall defects might compromise viability in the macrophages, with the failure to modulate phagosomal pH a secondary effect. There is some disagreement in the literature regarding the phenotypes of the *cwt1Δ* strain (37, 46), and further study will be required to dissect these possibilities. However, we note that there is precedent for a role for Cwt1 in carbon metabolism, as Sellam et al., found that it bound the promoters of a number of genes involved in the utilization of nonpreferred carbon sources (48).

During environmental neutralization induced by amino acid catabolism, the driving force behind the rise in pH is the excretion of ammonia derived from amino and side chain amine groups. We demonstrate here that no ammonia is released during growth on carboxylic acids, as expected since these compounds lack nitrogen. As a result, the chemical mechanism behind the rise in pH remains unclear. These compounds are acids, so their consumption may in itself contribute to the rise in pH, which closely tracks growth in the cultures, supporting this idea. Cells would, however, need some compensatory mechanism to maintain cytosolic pH balance. Part of this could be inherent in the metabolism of the acids: as glycolysis is acidogenic, gluconeogenesis consumes six protons for each glucose molecule generated. There could also be other basic compounds secreted into the medium. Metabolomic or other approaches will be needed to address this question.

While the metabolically driven mechanism we propose here and elsewhere (14, 24) is novel, remodeling of the phagosome is a common strategy of pathogens. The fungal pathogens *Cryptococcus neoformans* and *Histoplasma capsulatum* both appear to neutralize the phagosome by permeabilizing the organellar membrane via quite different routes (59–61). *C. glabrata* also occupies a neutral phagosome but, interestingly, cell viability is not required (62), in contrast to what we observe with *C. albicans*. A variety of bacterial pathogens also subvert phagosomal maturation and acidification, including *Mycobacterium tuberculosis*, *Legionella pneumophila*, *Listeria monocytogenes*, *Salmonella enterica*, and others (63–65). Our observations here represent the third separate mechanism—along with catabolism of amino acids and GlcNAc (14, 24, 28)—by which *C. albicans* can manipulate phagosomal maturation, indicative of the evolutionary pressures during the adaptation of this species to the mammalian host.

## MATERIALS AND METHODS

### Strains and media.

For routine propagation, *C. albicans* strains were grown in YPD medium (1% yeast extract, 2% peptone, 2% glucose, with or without 2% agar for solid or liquid medium) (66). Experiments in which the carbon source was varied utilized minimal YNB medium containing allantoin as the nitrogen source (YNBA; 0.17% yeast nitrogen base without amino acids or ammonium sulfate, 0.5% allantoin), with various compounds as the sole carbon source as indicated; the medium was adjusted to the starting pH (usually 4.0) using HCl (14, 27). The use of allantoin avoids the spontaneous generation of ammonia from ammonium sulfate seen in neutral- to basic-pH media (27). Selection for nourseothricin-resistant strains used YPD with 200 µg/ml of nourseothricin (YPD-Nou) (Werner Bioagents, Jena, Germany). The murine macrophagelike cell line J774A.1 was propagated in RPMI 1640 with glutamate, HEPES (HyClone), and 10% fetal bovine serum (FBS; VWR International) in a 5% CO_2_ environment.

The fungal strains used are listed in Table 3. The *cwt1Δ* and *cwt1Δ stp2Δ* deletion strains were generated using the SAT flipper methodology (49). Briefly, approximately 300 bp of homology immediately 5′ or 3′ of the *CWT1* ORF were amplified by PCR and cloned between the ApaI/XhoI and SacI/SacII sites, respectively, of pSFS2. The resulting *SAT1*-FLP cassette was used to transform *C. albicans* SC5314 and SVC17 (*stp2Δ*) strains by electroporation, with selection on YPD-Nou. Genomic DNA was isolated, and cassette integration confirmed in the selected candidates via PCR. Nourseothricin sensitivity was restored by inducing the expression of the Mal2p-FLP recombinase gene through growth on yeast extract-peptone-maltose medium. This process was repeated to generate the homozygous disruptants SVC36 (*cwt1Δ*::*FRT/cwt1Δ*::*FRT*) and SVC39 (*cwt1Δ*::*FRT/cwt1Δ*::*FRT stp2Δ*::*FRT/stp2Δ*::*FRT/stp2Δ*::*FRT*).

**TABLE 3:** *Candida* strains

Strain	Description or mutation	Complete genotype	Reference or source
SC5314	Wild type	Prototroph	75
SN250	Library control	*his1Δ*::*hisG*/*his1Δ*::*hisG leu2Δ*::*CdHis1*/*leu2Δ*::*CmLeu2 arg4Δ*::*hisG*/*arg4Δ*::*hisG*	38
SVC17	*stp2Δ*	*stp2Δ*::*FRT*/*stp2Δ*::*FRT*	14
MLC112	ATO1*	*ura3*/*ura3 RPS10*/*rps10*::CIp10*-ACT1p-ATO1(G53D)*	24
HDC31	*ato5Δ*	*ato5Δ*::*FRT*/*ato5Δ*::*FRT RPS10*/*rps10*::CIp10*-SAT1*	27
ACC2	*ach1Δ*	*ach1Δ*::*hisG*/*ach1Δ*::*hisG ura3*/*ura3 RPS10*/*rps10*::CIp10*-URA3*	76
SVC36	*cwt1Δ*	*cwt1Δ*::*FRT*/*cwt1Δ*::*FRT*	This study
SVC41	*cwt1+CWT1*	*cwt1Δ*::*FRT*/*cwt1Δ*::*FRT RPS10*/*rps10*::CIp10*-SAT1-CWT1*	This study
SVC39	*cwt1Δ stp2Δ*	*cwt1Δ*::*FRT*/*cwt1Δ*::*FRT stp2Δ*::*FRT*/*stp2Δ*::*FRT*	This study

Complementation of the mutant strain used plasmid pSV-7, a *SAT1*-marked version of CIp10 expressing *CWT1* under its native promoter. pSV-7 was generated by cloning the entire *CWP1* ORF into the ApaI and XhoI sites of pAG6 (14). This plasmid was linearized with StuI and used to transform *cwt1Δ* mutant cells to generate the *CWT1*-complemented strain (*RPS10/rps10*::CIp10*-CWT1-SAT1*).

### Alkalinization, morphology, and ammonia release assays.

The ability of strains to alter medium pH was assayed as described previously (14). Briefly, strains were grown overnight in YPD, washed in water, diluted to an initial optical density at 600 nm (OD_600_) of 0.2 in minimal YNB medium with the indicated carbon sources, and then incubated at 37°C in aerated cultures. At the indicated time points, the optical density and pH of the cultures were measured; in certain experiments, cellular morphology was assessed microscopically after fixation with 2.7% paraformaldehyde.

Ammonia generation was assayed as described previously (24). Overnight YPD cultures were washed and resuspended in water at an OD_600_ of 1.0 and then spotted (5 µl) onto solid YNBA medium with the indicated carbon source and incubated at 37°C. A small reservoir (the cap of a microfuge tube) was affixed to the lid of the petri dish directly opposite the newly developing colony and filled with 10% citric acid. The nitrogen content of this acid trap was determined using Nessler’s reagent as described previously (24). Experiments were performed at least in triplicate, and the results analyzed using Excel and Prism (GraphPad Software, Inc.).

### Genomic analysis of growth on alternative carbon sources.

To assess transcriptional profiles in cells exposed to different carbon sources, the wild-type SC5314 strain was grown overnight in YPD, washed, diluted to an OD of ~0.2 in minimal YNBA medium containing 2% glucose, 1% CAA, 10 mM glutamate, or 10 mM αKG, pH 4.0, and grown at 37°C until the pHs of alkalinizing cultures reached 5.2 to 5.5 (approximately 5 to 7 h). Cells grown in YNB medium with glucose were collected upon alkalinization of the cultures grown in YNB medium with CAA to pH 5.5. RNA was then prepared using a hot acidic phenol protocol (67), and RNA quality verified via rRNA integrity assayed on an Agilent 2100 Bioanalyzer. Illumina 454 sequencing was performed by Axeq, Inc., on poly(A)-selected samples. Raw sequence reads were mapped to assembly 21 of the *C. albicans* genome as annotated by the Candida Genome Database (68), using the Tuxedo suite of software (69–72) to generate the number of fragments per kilobase per million mapped reads (FPKM) for each gene. The sequence quality was very high and averaged 150× genome coverage (see Table S1 in the supplemental material).

Identification of differentially regulated gene sets was accomplished using an iterative approach using *K*-means clustering with Cluster and TreeView (73, 74). The coregulated clusters were analyzed for common biological functions using the GO term mapper at the Candida Genome Database.

### Genetic analysis of carboxylic acid-induced neutralization.

We obtained two homozygous mutant libraries from the Fungal Genetics Stock Center (Kansas City, MO), the Homann collection of transcription factor knockouts (37) and the broader Noble library (38), together comprising ~840 strains. These were propagated from the stock plates in 96-well plates in YPD medium overnight and then transferred to YNB with 10 mM αKG and 0.01% bromocresol purple, adjusted to pH 4.0. The medium also contained 40 µM arginine, as these strains are *arg4Δ*; the concentration was empirically determined as the lowest amount to support optimal growth. The plates were incubated at 37°C with aeration for 24 to 48 h and inspected visually for wells with color changes (due to the pH indicator) that deviated significantly from the controls. Most of the mutants were represented by two independently constructed strains, and both were tested. Candidate mutants were subjected to secondary screens in aerated liquid cultures for both growth and pH, as described above.

### Phagocytosis rate and hyphal formation of phagocytosed *C. albicans.*

To assess the interaction of *C. albicans* cells with the macrophages, we seeded 5 × 10^5^ cells of J774A.1 macrophages to glass coverslips in a 12-well plate and incubated them overnight at 37°C and 5% CO_2_. *C. albicans* cells were grown in YPD medium overnight, diluted 1:100 in YNB medium, and grown for 3 h at 30°C. Cells were then washed in distilled water (dH_2_O) and stained with 1 μM concanavalin A-FITC for 15 min, washed two times with phosphate-buffered saline (PBS), and resuspended in RPMI medium (HyClone). Amounts of 1 × 10^6^ *C. albicans* cells were cocultured with the macrophages at 37°C for 1 h. The cocultures were then washed twice with PBS, nonphagocytosed cells were stained with 35 μg/ml of Calcofluor white for 1 min, and the excess dye was removed by washing three times in PBS. Next, cells were fixed with 2.7% paraformaldehyde for 20 min at room temperature and washed with PBS. Images of the *Candida*-macrophage interaction were taken using an Olympus IX81 automated inverted microscope. Images from at least 100 phagocytosed cells per experiment were analyzed using SlideBook 6.0 software. The percentage of cells phagocytosed after 1 h of coculture was calculated using the following formula: (percent internalized cells/total number of cells) × 100. Hyphal morphogenesis during phagocytosis was quantitated by scoring the morphology of phagocytosed cells after 1 h of coculture using the following formula: (germ tubes + hyphal cells/total amount of cells) × 100. Experiments were performed in triplicate.

### Assessment of phagosomal pH.

Phagosomal pH was assessed as previously described (14, 27). Briefly, 1 × 10^6^ J774A.1 cells/ml were seeded onto glass coverslips in phenol red-free RPMI in 12-well plates and allowed to adhere for 2 h. Next, 50 nM LysoTracker red DM99 (Molecular Probes) was added to the cells and incubated for 2 h to ensure concentration of the dye in the lysosomes. *C. albicans* cells were grown overnight in YPD medium, diluted 1:100 in fresh YNB, and grown for 3 h at 30°C. Then, cells were washed in dH_2_O and stained with 1 μM concanavalin A-FITC for 15 min, and excess dye was removed by washing three times in PBS. Cells were then diluted to 1 × 10^6^ cells/ml in phenol red-free RPMI medium and cocultured with the macrophages for the indicated times at 37°C and 5% CO_2._ After washing with PBS to remove cell debris and nonadherent cells, the remaining cells were fixed in 2.7% paraformaldehyde for 20 min and stored at 4°C in PBS before visualization. The cocultures were imaged at ×60 magnification with an Olympus IX81 automated inverted microscope using the appropriate filter sets. To estimate the relative phagosomal pH, the signal intensities of both FITC and Lysotracker Red (LR) were plotted along a line drawn transversely across the short axis of the cell for at least 50 cells per condition using Slidebook 6.0 software. The average LR signal intensity was calculated for a region of 10 pixels (1 µm) immediately outside the fungal cell, whose boundary was determined by the slope of the FITC signal. All experiments were performed at least in triplicate.

### Assessment of fungal survival and macrophage cytotoxicity.

*C. albicans* survival during interaction with the J774A.1 macrophages was assessed as previously described (14). Macrophages were collected by centrifugation at 700 × *g* for 3 min, washed with PBS, and resuspended in fresh RPMI medium. Cells were seeded at 2.5 × 10^4^ cells/well in 96-well plates and grown overnight at 37°C and 5% CO_2_. Log-phase *C. albicans* cells were washed in dH_2_O and resuspended in fresh RPMI medium. Amounts of 1 × 10^4^ cells/well were added to wells containing macrophages or medium alone, followed by six serial 1:5 dilutions. After 48 h at 37°C and 5% CO_2_, microcolonies of *C. albicans* were counted, using an inverted microscope, in wells in which individual colonies could be distinguished. The results were presented as the following ratio: (number of colonies in the presence of macrophages/number of colonies without macrophages) × 100. The experiment was performed in triplicate.

*C. albicans* toxicity on macrophages was assessed using the CytoTox96 nonradioactive cytotoxicity assay (Promega) as previously described (14). Briefly, J774A.1 macrophages were prepared as described above, seeded at 2.5 × 10^5^ cells per well in a 96-well plate, and incubated overnight at 37°C and 5% CO_2_. *C. albicans* cells were grown to log phase in YNB medium, washed in PBS, and cocultured with macrophages at a 3:1 ratio for 5 h. To assess macrophage cytotoxicity, the plates were centrifuged at 250 × *g* for 4 min and 50-μl aliquots of the coculture supernatant were transferred to a fresh plate and mixed with an equal volume of substrate mixture. After 30 min of incubation, the reaction was stopped with 50 μl of Stop solution and the absorbance at 490 nm recorded. The data from spontaneous release of lactate dehydrogenase (LDH) by the macrophages and by the *C. albicans* cells alone, as well as maximum LDH release from lysed macrophages, were used to calculate *C. albicans* cytotoxicity on macrophages according to the manufacturer’s protocol. The experiment was performed in triplicate.

### Accession number(s).

The RNA-seq data set is available through the Gene Expression Omnibus database (https://www.ncbi.nlm.nih.gov/geo) under GenBank accession number GSE87832.

## SUPPLEMENTAL MATERIAL

Figure S1  Transcriptional analysis shows a comprehensive shift to gluconeogenesis. The fold change in expression for each gene is given relative to its expression in glucose for the wild-type SC5314 strain grown in α-ketoglutarate, Casamino Acids, or glutamate. The redirection of carbon flows toward gluconeogenesis is readily apparent. Download Figure S1, PDF file, 0.1 MB

Figure S2  Mutants with defects in growth and pH neutralization on αKG. Strains from the Homann and Noble libraries were grown at 37°C in YNB with 10 mM αKG and 40 µM arginine for 24 h. Growth is plotted on the left axis (black bars), and culture pH on the right axis (grey bars). The changes in pH are significant (*P* < 0.05) for each of the mutants relative to the result for the control strain (SC5314). Download Figure S2, PDF file, 0.1 MB

Table S1  RNA-sequencing statistics. Data regarding the transcriptional profiling via Illumina deep sequencing are given. Table S1, PDF file, 0.1 MB
