# Supplementary figures and images for: Robust Extracellular pH Modulation by Candida albicans during Growth in Carboxylic Acids
Source: mBio. 2016 Nov 15;7(6):e01646-16. doi: 10.1128/mBio.01646-16 (PMC5111404; doi:10.1128/mBio.01646-16)

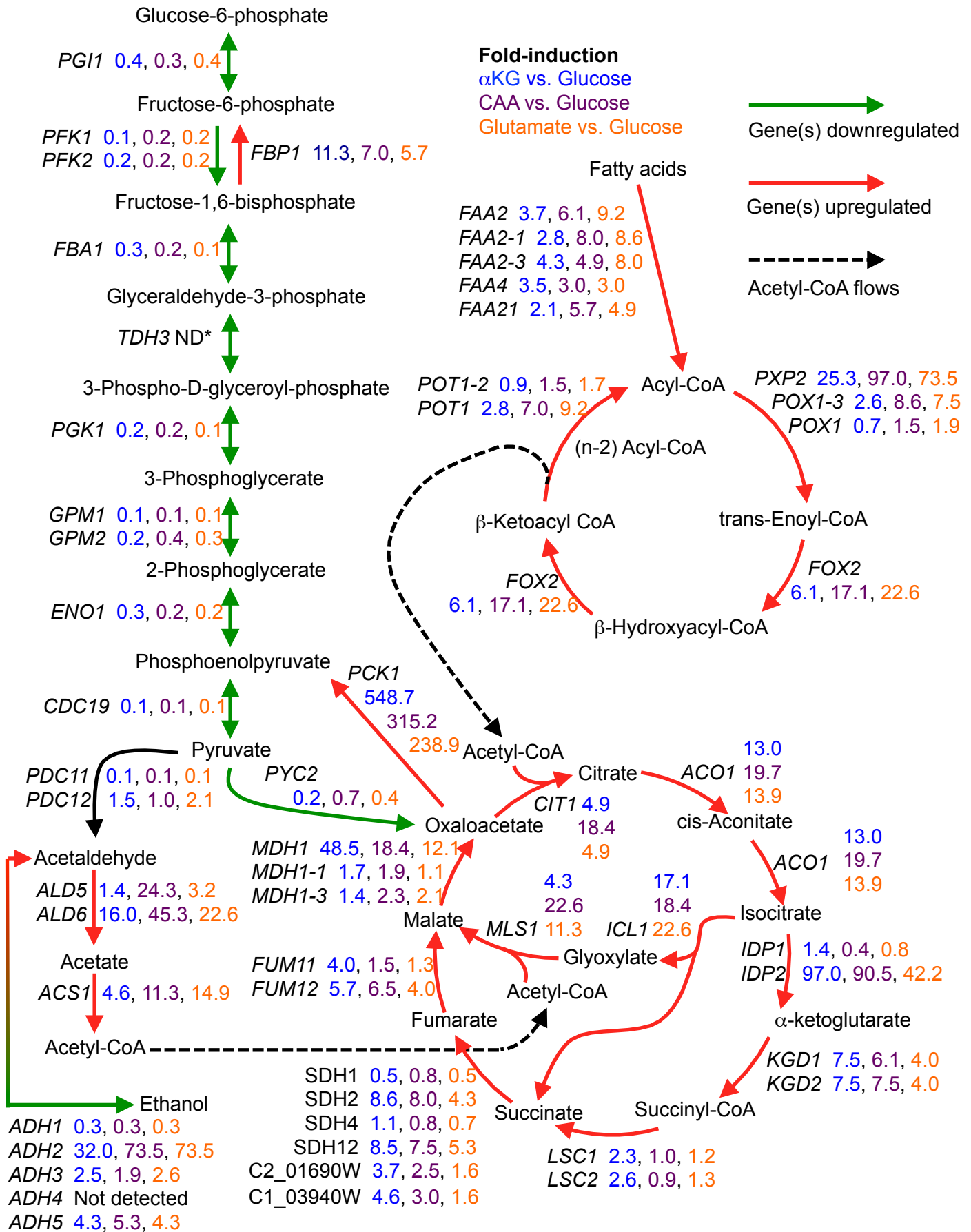

Supplement: Figure S1 — Transcriptional analysis shows a comprehensive shift to gluconeogenesis. The fold change in expression for each gene is given relative to its expression in glucose for the wild-type SC5314 strain grown in α-ketoglutarate, Casamino Acids, or glutamate. The redirection of carbon flows toward gluconeogenesis is readily apparent. Download [file mbo006163074sf1.pdf]

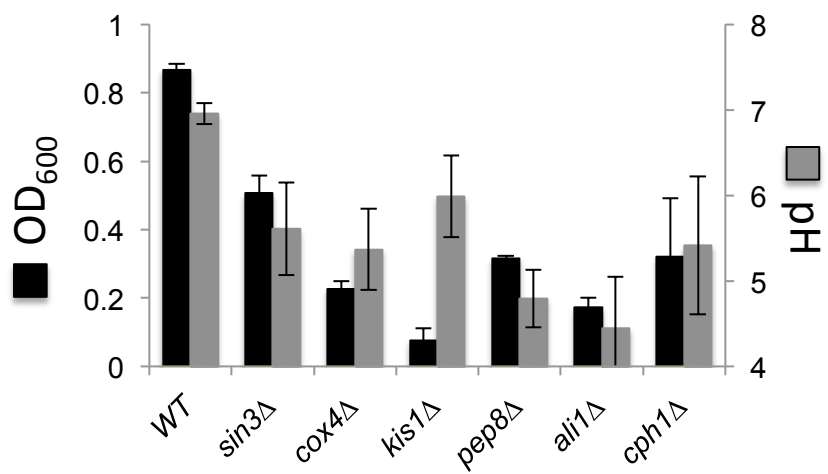

Supplement: Figure S2 — Mutants with defects in growth and pH neutralization on αKG. Strains from the Homann and Noble libraries were grown at 37°C in YNB with 10 mM αKG and 40 µM arginine for 24 h. Growth is plotted on the left axis (black bars), and culture pH on the right axis (grey bars). The changes in pH are significant (P < 0.05) for each of the mutants relative to the result for the control strain (SC5314). Download [file mbo006163074sf2.pdf]
