# Supplementary material for: Robust Extracellular pH Modulation by Candida albicans during Growth in Carboxylic Acids
Source: mBio. 2016 Nov 15;7(6):e01646-16. doi: 10.1128/mBio.01646-16 (PMC5111404; doi:10.1128/mBio.01646-16)
Supplement: Table S1 — RNA-sequencing statistics. Data regarding the transcriptional profiling via Illumina deep sequencing are given. [file mbo006163074st1.pdf]

Supplemental Table 1. RNA sequencing statistics

Carbon Source[1]	Replicate	Reads (million)	N (%)[2]	Q20 (%)[3]	GC (%)	Genome coverage[4]
Glucose	1	46.9	0.071	96.2	36.6	166x
Glucose	2	41.5	0.070	96.1	36.5	147x
CAA	1	42.6	0.071	96.2	35.9	151x
CAA	2	49.5	0.071	96.5	35.8	175x
Glutamate	1	44.1	0.069	96.5	36.1	156x
Glutamate	2	39.3	0.072	96.6	35.3	139x
αKG	1	47.2	0.071	96.2	36.1	167x
αKG	2	43.1	0.071	96.5	35.8	152x

1. The media was minimal YNB with 0.5% ammonium sulfate and 2% of the indicated compound.
2. Percentage of uncalled bases.
3. Percent of reads at Q20 (~99% accurate base calling) or higher.
4. Based on a diploid genome size of 28.6 Mbp (per Candida Genome Database).
